# Multi-slice frozen phonon simulations of high-angle annular dark field scanning transmission electron microscopy images of the structurally and compositionally complex Mo–V–Nb–Te oxide catalyst

**DOI:** 10.1186/s40679-018-0058-5

**Published:** 2018-07-31

**Authors:** Douglas A. Blom, Thomas Vogt

**Affiliations:** 1 NanoCenter and Department of Chemical Engineering, University of South Carolina, Columbia, SC 29201 USA; 2 NanoCenter and Department of Chemistry & Biochemistry, University of South Carolina, Columbia, SC 29201 USA

**Keywords:** Frozen phonon multi-slice, M1 catalyst, Quantitative HAADF-STEM

## Abstract

We report frozen phonon multi-slice image simulations for the complex oxidation catalyst M1. Quantitative analysis of the simulations suggests that the detailed order of the cations along the electron propagation direction in a [001] zone axis orientation can lead to different high-angle annular dark field signals from atomic columns with identical composition. The annular dark field signal varies linearly with atomic percent V, and the spread of intensities due to the atomic species order is of similar magnitude to the intensity difference due to ± 5% V.

## Background

**Fig. 1:**
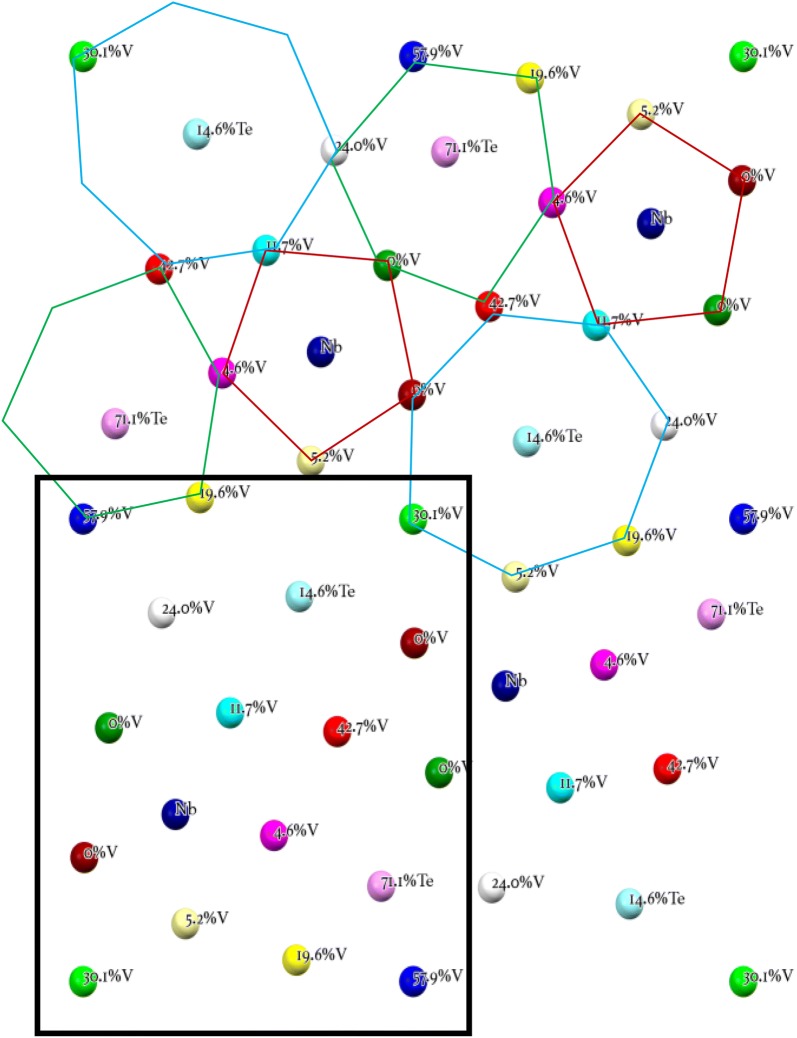
Cation positions in the [001] projection of the MoVNbTe oxide M1 catalyst. Site colors correspond to crystallographically distinct cation sites. The black rectangle illustrates the size of the area simulated. The pentagonal (dark red), hexagonal (green) and heptagonal (light blue) rings of metal–oxygen octahedra are outlined above. Unit cell parameters [4]: Pba2 (no. 32); *a* = 2.1134(1) nm, *b* = 2.6647(1) nm, *c* = 0.40140(2) nm

Annual worldwide acrylonitrile (ACN) production is nearly 1 kg/person [1]. Currently, production uses the “SOHIO process” originally developed in the 1950s [2]. Propylene is reacted over a multi-phase catalyst in the presence of ammonia. For both economic and energy use reasons, there is a strong desire to produce acrylonitrile using propane as starting material. This requires the development of a new catalyst. The most promising catalyst for the direct ammoxidation of propane to acrylonitrile is a quaternary Mo–V–Nb–Te oxide phase originally reported by the Mitsubishi Chemical Co., and therefore, typically referred to in the literature as “M1”. The crystal structure was originally solved with the combined Rietveld refinement of synchrotron X-ray and neutron powder diffraction data [3]. More recently, the model was improved [4] due to input from high angle annular dark field (HAADF) scanning transmission electron microscope (STEM) observations [5]. Figure 1 reveals the structural model of the cation positions in this phase in a [001] orientation. The colors in the figure correspond to the crystallographically distinct cation columns. The unit cell consists of a series of pentagonal, hexagonal and heptagonal rings of metal–oxygen octahedral and is one octahedron thick along the *c*-axis. The pentagonal ring is dark red, the hexagonal ring is green and the heptagonal ring is light blue in Fig. 1. The center of the pentagonal ring is occupied by Nb. Te and oxygen chains are present in both the hexagonal and heptagonal channels. The other ten crystallographically distinct cation sites are populated by various mixtures of Mo and V as shown in Fig. 1.

HAADF STEM has become a common characterization technique for M1 and a number of isostructural phases with various elemental compositions [6–10]. The initial work in [5] was followed by analysis of M1 phases with different synthesis conditions [6]. The primary difference was found to be in the Te–O chains. The framework of the phases was unaffected by the change in the synthesis type. By replacing the Nb with Ta, HAADF STEM was able to demonstrate that the Ta was incorporated into the center of the pentagonal unit both for an ambient pressure “slurry” synthesis [7] and a hydrothermal synthesis [9] suggesting that the Nb in the traditional M1 phase will also be incorporated into the center of the pentagonal unit as originally suggested by the Rietveld refinement model of [3].

In addition to the isostructural phases, HAADF STEM has been used to characterize a number of compositionally similar phases with a variety of related structures [11, 12], intergrowths of several of these phases [13, 14] as well as the initial formation of nascent crystallites [15]. The effects of heating [16] and in situ heating, and gas exposure on the structure and composition of M1 phases [17, 18] have been studied using HAADF-STEM. In all of these reports, the analysis of the STEM data was focused on either the spatial location of the atomic columns in the [001] orientation or a qualitative analysis of the intensity of the HAADF-STEM images using an incoherent imaging model.

The propane ammoxidation activity and selectivity of the M1 catalyst and a number of chemically and structurally related oxide phases varies widely in the literature depending on a number of factors which are not fully understood [19]. One of the proposed catalytic mechanisms suggests that the local distribution of V^5+^ ions on the surface is an important parameter to achieve simultaneously a high conversion rate for propane and high selectivity to the desired ACN product [20]. Recently, He et al. [14] reported on the design of a mesoscale intergrown catalyst of M1 and M2, providing clear evidence that this proposed mechanism is correct. Therefore, the spatial distribution of V in these catalysts may be key to understanding and hopefully optimizing their performance.

The potential ability of Z-contrast STEM to provide localized information regarding the mass thickness of a specimen is uniquely suited to characterizing the V distribution in these materials. Multi-slice image simulations will be required to make quantitative comparisons between HAADF STEM images and atomic column compositions. Recently, Epicier et al. [21] reported on the spatial distribution of V using a combination of HAADF STEM and multi-slice image simulations. They found that their images followed a power-law relationship between mass thickness and image intensity. For the closely related MoVTeTaO system, Woo et al. [22, 23] performed quantitative analysis of HAADF-STEM images to deduce the V distribution. The two reports do not agree regarding the relationship between the STEM image contrast and atomic column composition. Both groups reported V concentrations derived from measurements of HAADF STEM images to a precision of 1% V or better. Neither report addressed the role the order of the Mo and V atoms in an atomic column will have on the STEM image intensity. Heidelmann et al. [10] have recently published data on a closely related compound, Cs_0.44_[Nb_2.54_W_2.46_O_14_], demonstrating a linear relationship between W content and ADF STEM intensity for a variety of thicknesses. They reported that the ordering of the Nb and W atoms in the column produced as much as a 10% change in simulated ADF image intensities.

Previously, we have published results of multi-slice ADF STEM image simulations on this material [24, 25]. The virtual crystal approximation (VCA) was used in the published simulations. This means that the projected potential of the cation sites with partial substitution of Mo and V in the VCA is the weighted average of the potentials of Mo and V regardless of their order. For STEM, the ADF signal is sensitive to not only the average mass of the cations but also the location of the cations along the beam propagation direction primarily due to the effect of electron channeling [26]. In this work, we report the results of multi-slice ADF STEM image simulations based on the improved structural model [4] without using the VCA.

## Methods

A number of simulations were performed in which the order of the cations along the beam direction was varied while maintaining an overall stoichiometry consistent with the refined crystallographic model for each cation site. A random solid solution was assumed in building these models. As a compromise between CPU time requirements and ability to simulate closely related V compositions, a sample thickness of 30 cations along <001> was chosen. A random number generator was run 30 times for each cation site to generate a list of V and Mo cations consistent with the model structure occupancy or in the case of the Te-intercalated sites, a list of occupied and unoccupied Te–O chains. The input structure file for the multi-slice image simulations was a list of 4757 atom positions and thermal parameters.

**Fig. 2:**
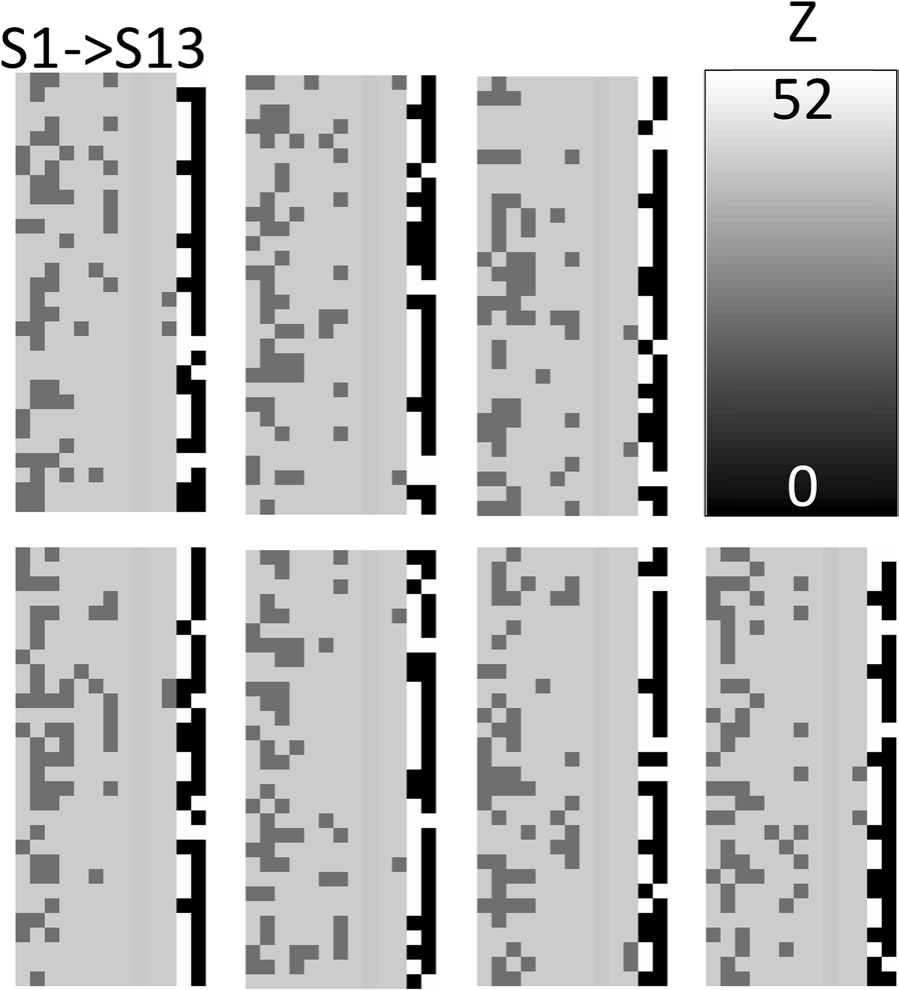
Graphical representation of the seven distinct configurations simulated in this study. Atomic sites S1–S13 from left to right. Each column corresponds to a particular cation site in the structure, while the intensity is equal to the atomic number of the cation. The last two columns on the right correspond to the Te–O chains in the hexagonal and heptagonal sites, respectively. The stoichiometry of each cation site is identical for all the configurations, while the location of the V substitution varies for each. A random solid solution was assumed in building these model configurations

Frozen phonon simulations using the multi-slice code of Kirkland [27] were performed using the Extreme Science and Discovery Environment (XSEDE) Stampede at the Texas Advanced Computing Center through allocation TG-DMR120079 [28]. An accelerating potential of 200 kV, convergence semi-angle of 17.3 mrad, a spherical aberration constant Cs of 3 µm and a defocus of 2 nm were the microscope parameters and chosen to match those of our published HAADF STEM of M1 [5–8, 12, 13, 15]. The ADF detector in the simulation spanned 100–425 mrad. Sampling convergence tests were performed on the size of the super-cell, array sizes for the transmission function and probe function and the slice thickness [24]. A super-cell of 8.453 by 7.994 nm (4*a *× 3*b*) was sampled by a transmission function array size of 6000 × 6000. The probe array size was 1800 × 1800 and stepped at 0.0100594 nm per pixel. The slice thickness was set at *c*/3 (0.1338 nm). The bandwidth limited maximum scattering considered in these simulations is 593 mrad. 64 phonon passes were necessary to achieve convergence of the simulations [24]. The simulations were carried out line by line for an area slightly larger than 1/4 of the unit cell which contained all the cation sites of the structure (see Fig. 1) for a total of 163 lines of 135 pixels each. The simulated thickness was 30 unit cells along the *c*-axis (12 nm), which allows for 30 different cations along the electron propagation direction. The atomic coordinates, isotropic thermal parameters, and occupancy were taken from [4]. Because of the finite number of cations, the composition at each site was quantized in 3.3% substitutional increments. Table 1 lists the refined V concentrations for the various cation sites, the V content used in the image simulations and the possible number of different atomic configurations for each cation site consistent with the composition. For example, cation site S1 has a composition of 30% V, or 9 V atoms and 21 Mo atoms. There are 30!/9!21! different ways to arrange this collection of atoms. Each simulated configuration required 1.1 CPU years to complete, precluding the calculation of more cation distributions. Figure 2 is a graphical representation of the cation distributions along the beam direction for the 13 independent cation sites from S1 to S13 from left to right. S9 contains the Nb in this model and S12 and S13 are the intercalated Te–O sites. The intensity is equal to the atomic number of the cation for all 30 possible locations. The last two columns are the Te-containing sites that are either vacant or contain Te–O chains.

**Table 1:** Possible combinations for Mo/V mixed cation sites, V content from the model structure, and input for the multi-slice simulations

Cation site	Combinations	V concentration (%)	
		Model structure [4]	Image simulation
S1	14307150	30.1	30
S2	119759850	57.9	56.$\bar{6}$
S3	86493225	42.7	40
S4	593775	19.6	20
S5	30	4.6	3.$\bar{3}$
S6	4060	11.7	10
S7	2035800	24.0	23.$\bar{3}$
S8, S10	1	0	0
S11	435	5.2	6.$\bar{6}$

## Results and discussion

**Fig. 3:**
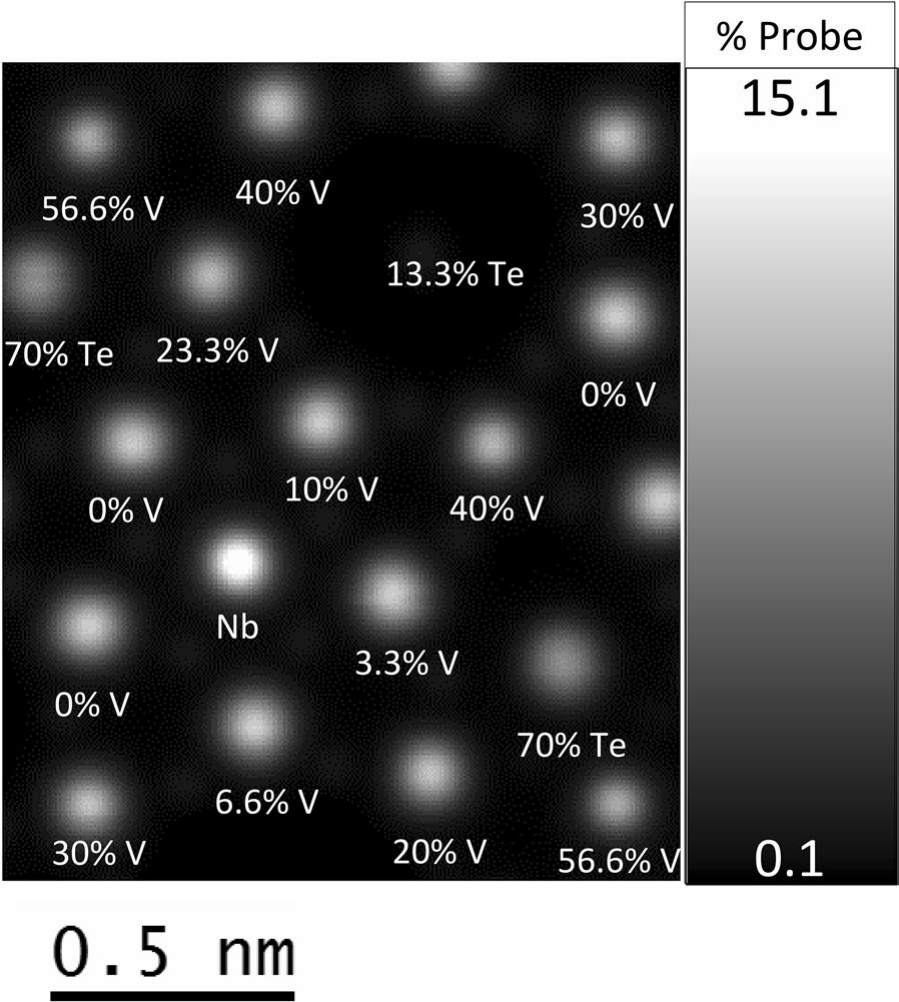
Image simulation result for one of the configurations. Cation columns are labeled with their composition. Intensity scale is in percent of initial probe

Figure 3 shows the simulation results of one of the atomic configurations. The image displayed is in units of percentage of the initial probe current and varies between 0.1 and 15.1%. In the simulation, there are multiple versions of sites S1, S2 and S10. Only one was used for the data analysis. Even though Nb is slightly lighter than Mo, the Nb column appears much brighter than either of the two fully Mo-occupied sites, due to the much smaller thermal parameter for the Nb site in the model structure. The Te–O chains inside the heptagonal channels scattered 1.5% of the initial probe current according to the simulations and are not visually apparent. The Te–O chains in the hexagonal channels are 70% filled in the simulation, scattered almost 11% of the initial probe current and are clearly visible.

**Fig. 4:**
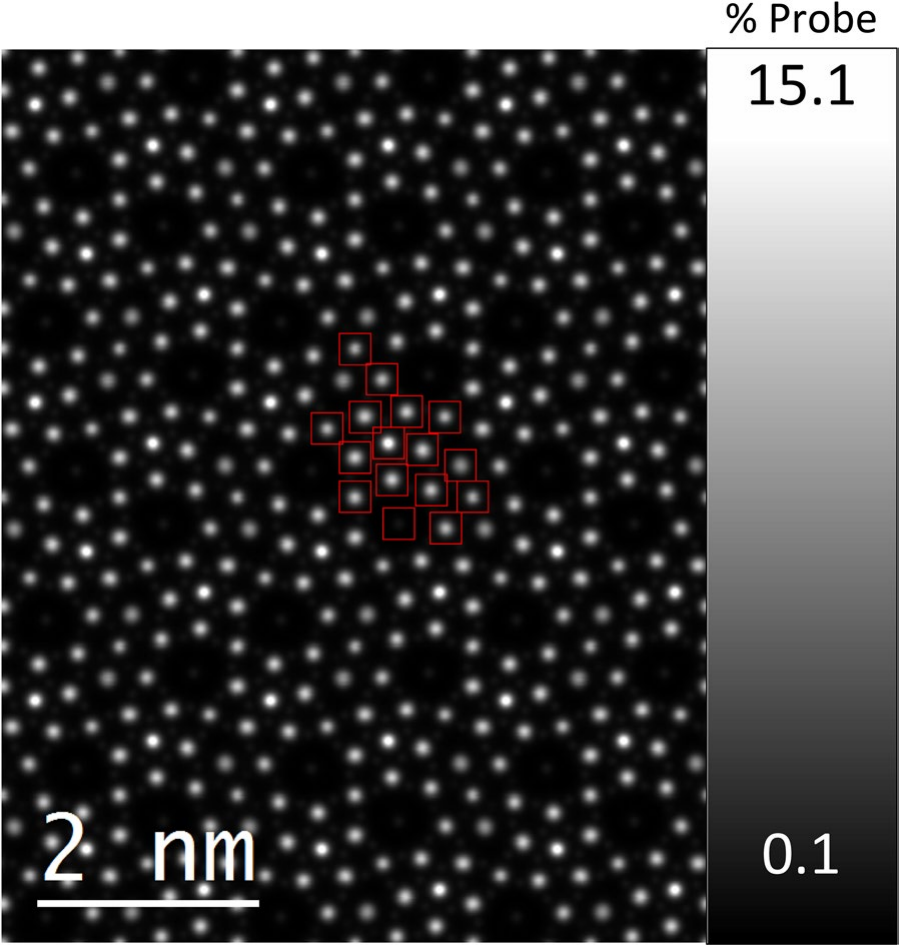
3*a *× 3*b* tiled image simulation results. The regions of interest used in the image quantification are shown as red boxes. Intensity scale is in percent of initial probe

The regions of interest (ROIs) used to extract quantitative ADF image intensity values for the M1 cation sites are shown as red boxes in Fig. 4. Figure 4 corresponds to a 3*a *× 3*b* projected area produced by appropriately tiling the image simulation results using the symmetry of the unit cell. Intensity scale is identical to Fig. 3. Quantification of the HAADF image intensity was performed by integrating the HAADF signal inside each ROI.

**Fig. 5:**
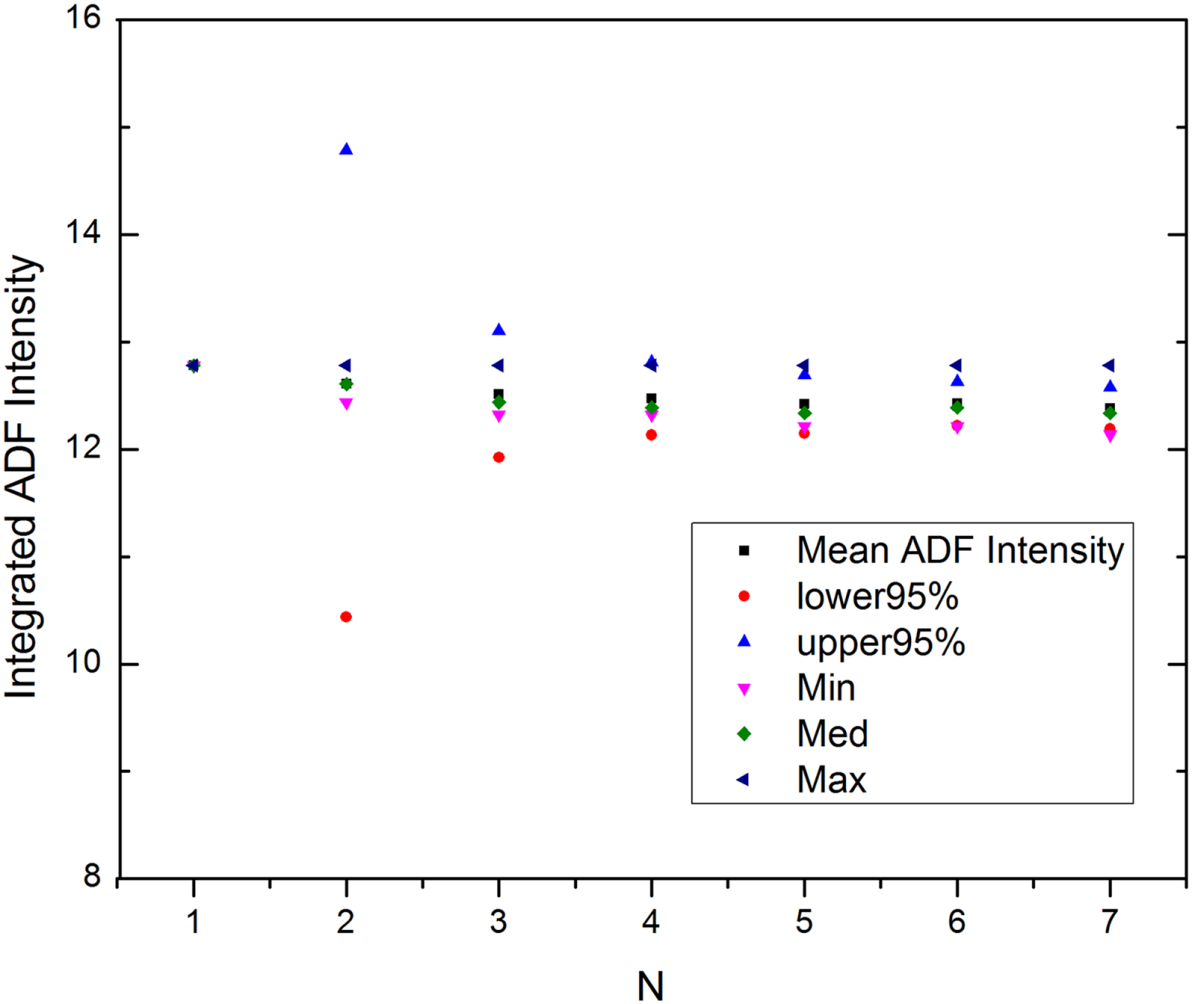
Student’s *t* distribution 95% confidence limits for cation site S3 (40% V) as a function of the number of atomic configurations. The minimum, mean, median, and maximum values are also plotted

Figure 5 shows the variation of the HAADF intensity due to the distribution of Mo and V along [001] for cation site S3 (40% V). For the configurations considered, the Student’s *t* distribution 95% confidence limits of the mean intensity become relatively narrow after only four different configurations. The additional configurations make only a small difference, suggesting that the detailed order of Mo and V along the beam direction plays a relatively minor role in the integrated HAADF intensity for the configurations considered here. The other cation sites exhibited a similar behavior, leading us to confine ourselves to seven different atomic configurations.

**Fig. 6:**
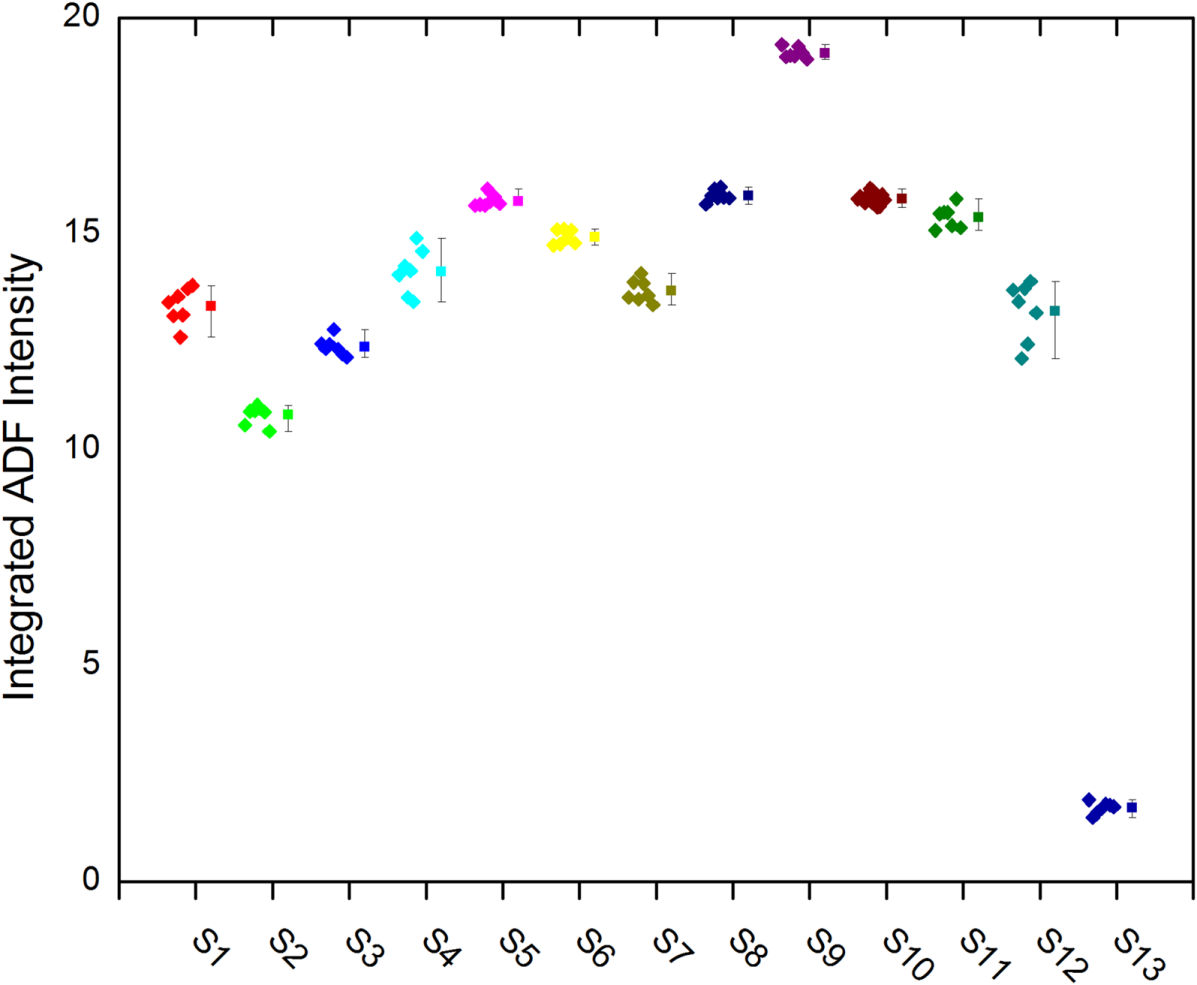
Box plot of the ADF intensities for the 13 cation sites in M1. The data for each of the seven configurations is shown to the left of the mean and 95% confidence limits

Figure 6 is a box plot for all 13 cations sites in M1. The individual data points are shown to the left of the mean and 95% Student’s *t* distribution confidence limits. Sites 8 and 10 are fully Mo occupied and site 9 is 100% Nb. The narrow confidence limits for these sites indicates that the sampling of the simulations (real and reciprocal space) and number of phonon passes is sufficient for high accuracy in the simulations. Sites S12 and S13 are the intercalated Te–O sites.

**Fig. 7:**
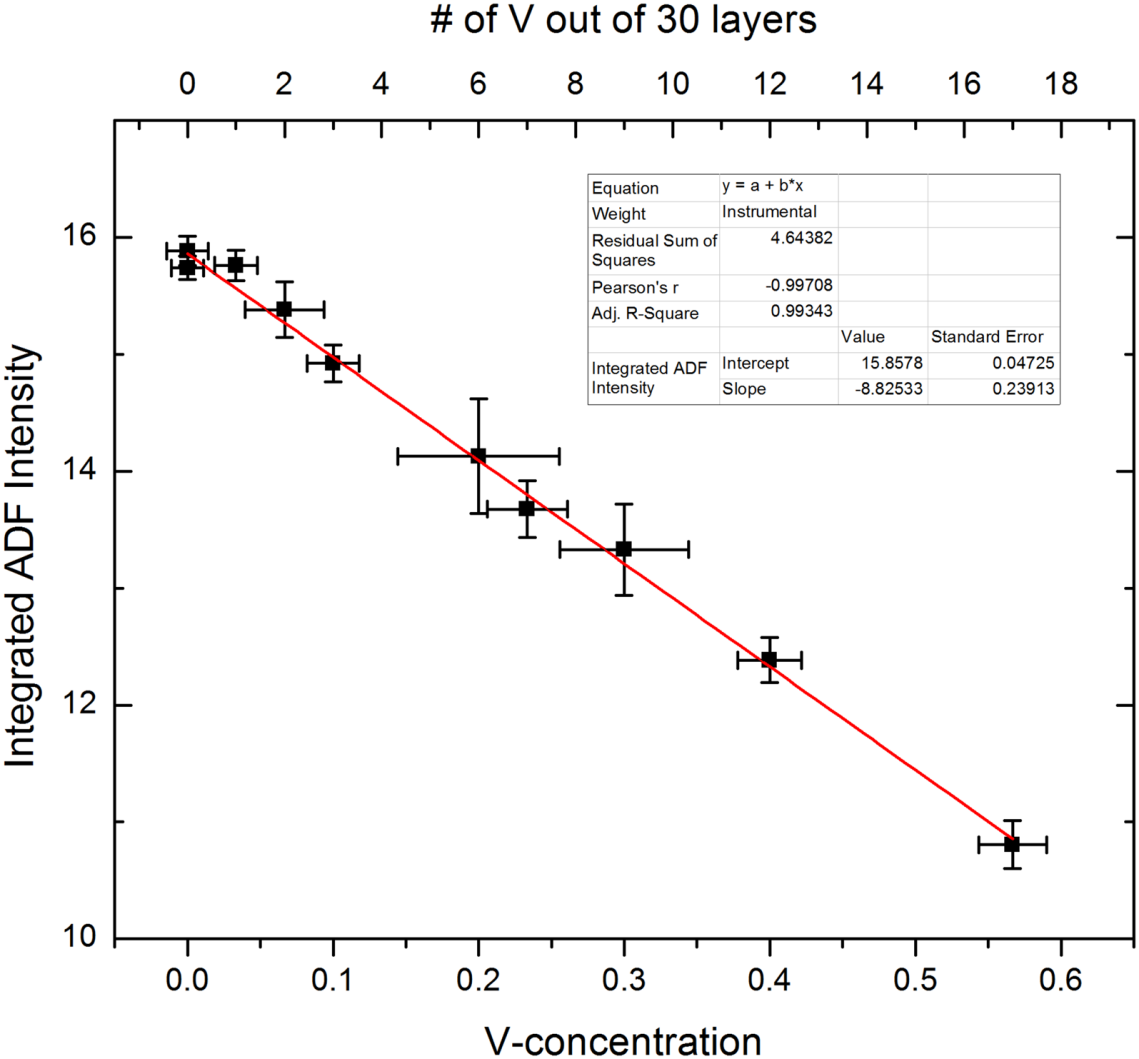
V content vs. ADF intensity for M1. Error bars are the 95% confidence limits on the mean given the range of intensities measured from the seven different atomic configurations considered here

Figure 7 plots the mean-integrated HAADF intensity as a function of the V content. Symbols mark the mean. The *Y* error bars are the 95% confidence limits. The red line is a linear regression to the means. The mean ADF intensity in M1 is well described by a linear change in intensity with V content for the conditions considered here. The *X* error bars are the apparent uncertainty in V content from the intensity confidence limits and the slope of the regression. In general, the variability due to order along the beam propagation direction is ≈ ± 1 atom of V out of the 30 unit cells in these simulations. In other words, the detailed ordering of the Mo and V along the columns for an identical composition leads to changes in ADF image intensity equivalent to an uncertainty in the composition of ± 3.3 atomic % V. In the worst cases, the uncertainty in the V concentration is equivalent to ± 2 atoms (i.e., ± 6.6% V). Given these results, it seems unlikely that quantitative HAADF STEM of M1 will be able to determine the V content in an atomic column to the precision previously reported [21–23].

These image simulations have not considered many of the artifacts observed in experimental quantitative HAADF STEM. Incoherent blurring of experimental HAADF STEM images is a well-known phenomenon. By integrating the output of the multi-slice HAADF STEM image simulations, these incoherent effects will be less important. Electron dose sensitivity will add an additional uncertainty in the quantification of experimental HAADF images, as will amorphous surface layers and carbon contamination. While other experimental difficulties need to be considered, these image simulation results suggest that for Mo/V-substituted atomic columns, the order of the cations along the beam propagation direction do contribute to measureable differences in integrated HAADF signals from columns of identical composition, limiting the precision of assigning a composition to any particular atomic column.

## Conclusions

Our frozen phonon multi-slice HAADF image simulations have shown that for atomic columns with alternating O and either Mo or V, as found in MoV bronzes, the sequence along the electron beam direction has a relatively minor effect on the ADF intensity at the atomic column location. The mean ADF intensity varies linearly with V content up to 56% V. However, the uncertainty of the V concentration does not vary linearly but peaks for compositions near 25% V for the limited number of atomic configurations considered. Based on these simulations, quantitative ADF STEM may at best be able to distinguish atomic columns which have a V content that is 5% different for the limited set of conditions considered here. Other instrumental parameters or sample thickness may have different quantitative results. Regardless, the VCA is not appropriate to apply to multi-slice image simulations of the M1 phase. Image simulations which consider different ordering of cations in the sample matching experimental conditions should be considered before making quantitative statements assigning V content based solely on HAADF STEM image data.

## Abbreviations

ACN: acrylonitrile
HAADF: high-angle annular dark field
ROI: region of interest
STEM: scanning transmission electron microscopy
VCA: virtual crystal approximation
XSEDE: extreme science and discovery environment
